# Macrophage Transactivation for Chemokine Production Identified as a Negative Regulator of Granulomatous Inflammation Using Agent-Based Modeling

**DOI:** 10.3389/fimmu.2018.00637

**Published:** 2018-03-27

**Authors:** Daniel Moyo, Lynette Beattie, Paul S. Andrews, John W. J. Moore, Jon Timmis, Amy Sawtell, Stefan Hoehme, Adam T. Sampson, Paul M. Kaye

**Affiliations:** ^1^Centre for Immunology and Infection, Department of Biology and Hull York Medical School, University of York, York, United Kingdom; ^2^Department of Computer Science, University of York, York, United Kingdom; ^3^Department of Electronics, University of York, York, United Kingdom; ^4^SimOmics Ltd., York, United Kingdom; ^5^Institute for Computer Science, University of Leipzig, Leipzig, Germany; ^6^Division of Computing and Mathematics, Abertay University, Dundee, United Kingdom

**Keywords:** kupffer cells, granulomas, inflammation, *Leishmania*, natural killer T cells, agent-based modeling, computational immunology, liver

## Abstract

Cellular activation *in trans* by interferons, cytokines, and chemokines is a commonly recognized mechanism to amplify immune effector function and limit pathogen spread. However, an optimal host response also requires that collateral damage associated with inflammation is limited. This may be particularly so in the case of granulomatous inflammation, where an excessive number and/or excessively florid granulomas can have significant pathological consequences. Here, we have combined transcriptomics, agent-based modeling, and *in vivo* experimental approaches to study constraints on hepatic granuloma formation in a murine model of experimental leishmaniasis. We demonstrate that chemokine production by non-infected Kupffer cells in the *Leishmania donovani*-infected liver promotes competition with infected KCs for available iNKT cells, ultimately inhibiting the extent of granulomatous inflammation. We propose trans-activation for chemokine production as a novel broadly applicable mechanism that may operate early in infection to limit excessive focal inflammation.

## Introduction

Immune responses are commonly initiated by localized infectious insult and multiple mechanisms have evolved to allow spread of host effector responses to meet the challenge of pathogen containment. In the late 1950s, seminal studies by Isaacs and Lindenmann defined how “interferons” amplified local cellular resistance following virus infection (1, 2). A decade later, Mackaness described cross protective cellular immunity mediated *via* T cell cytokine-dependent macrophage activation (3). More recently, cytokine- and chemokine-mediated amplification of host protective immunity has been described across a spectrum of responses driven by both innate lymphoid cells and *via* conventional T cells (4–9). While serving to eliminate pathogens more effectively, a potentially undesirable consequence of amplifying immune effector responses is immunopathology, collateral damage induced by an overzealous drive toward inflammation. Hence, an equally impressive array of “regulatory” or “suppressive” mechanisms have been defined that serve to limit immunopathology, and that suggest an evolutionary balance between pathogen elimination and host survival (10–12).

Granulomatous inflammation represents an extreme form of focal inflammation, often initiated around pathogens or foreign bodies that pose a formidable challenge for immune clearance. Granulomas are a hallmark of the immunopathology of many human infectious diseases including tuberculosis (13, 14), schistosomiasis (15), and leishmaniasis (16). While granuloma formation may provide means for containment and be host beneficial, excessive granuloma formation, numerically or in terms of individual granuloma size can lead to severe pathological consequences. Hence, mechanisms for limiting the exuberance of the granulomatous response through late acting regulatory pathways are also well described in the literature (17–20). However, the question of whether additional regulatory mechanisms operate at the earliest stages of granuloma initiation and prevent or limit over-exuberant granuloma formation has not been previously addressed.

Experimental visceral leishmaniasis, resulting from infection of mice with the Kupffer cell (KC) tropic parasite *Leishmania donovani*, has provided a highly tractable tool to study the initiation of granulomatous pathology in the hepatic microenvironment. Following infection of mice with *L. donovani*, infected KCs transiently release the chemokines CCL1, CCL2, and CXCL10 in a T cell-independent manner, whereas sustained expression of CXCL10 is dependent upon IFNγ production by invariant NKT (iNKT) cells (21). IFNγ production by iNKT cells is in turn costimulated by ligation of CD47 on natural killer T (NKT) cells by signal regulatory protein alpha (SIRPα) expressed on KCs, providing positive feedback for sustained iNKT cell recruitment and KC activation (22). A similar role for CXC chemokines in recruiting hepatic NKT cells has been observed in other models of liver infection/inflammation (23, 24). For example, CXCL9 produced by KCs following infection with the bacterium *Borrelia burgdorferi* results in CXCR3-dependent clustering of NKT cells around infected KCs (25), whereas CXCR6 and its ligand CXCL16 regulate NKT cell accumulation in the liver during fibrosis (26). Hence, early recruitment of “amplifier” cells such as NKT cells is a central and common theme of focal inflammation.

Examination of the kinetics of granulomatous inflammation in this model of visceral leishmaniasis suggests, however, that there may be inherent limitations imposed on the ability of the host to form hepatic granulomas. Notably, granuloma formation proceeds asynchronously, and even many weeks after infection, fully formed granulomas sit side by side with infected KCs that appear to have failed to stimulate an inflammatory focus (16, 27). Here, we have combined transcriptional profiling and computational modeling to probe possible mechanisms that might underpin the asynchronous development of granulomas in this model. We demonstrate that KC chemokine production, contrary to expectations, is not restricted to infected cells alone, but spreads in trans to include uninfected KCs within the infected liver. Data generated using a novel agent-based model (ABM) in which KCs and iNKT cells interact within a spatially constrained sinusoidal network suggest that the spreading of chemokine production to uninfected KCs limits the competitiveness of infected KCs in terms of their ability to attract iNKT cells and initiate granuloma formation. *In silico* experiments predicted that this competition could be overcome by increasing the number of available NKT cells, a prediction borne out *in vivo*. Hence, our data identify a new pathway that operates early in infection to limit excessive inflammation by introducing competition for a finite resource (i.e., iNKT cells) that is needed for granuloma initiation.

## Materials and Methods

### Mice and Parasites

C57BL/6 mice were obtained from Charles River (UK). mT/mG (28) and LysMcre (29) mice have been previously described. Mice were bred and housed under specific pathogen-free conditions and used at 6–12 weeks of age. The tandom Tomato fluorescent protein expressing Ethiopian strain of *L. donovani* (tdTom.LV9) (30) was maintained by serial passage in *Rag1*^−/−^ mice. Amastigotes were isolated from infected spleens, and mice were infected with 3 × 10^7^ *L. donovani* amastigotes intravenously (i.v.) *via* the tail vein in 200 µl of RPMI 1640 (GIBCO, UK). All animal procedures were approved by the University of York Animal Welfare and Ethical Review Board and carried out under UK Home Office license (PPL 60/4377).

### Microarray Analysis

As previously described (31), KCs were flow sorted (on the basis of SSC/FSC and expression of CRIg, Gr-1 and F4/80) from naive mice and from infected mice and KCs from infected mice were further sorted (on the basis of TdTomato expression) into those containing amastigotes (“infected”) and those that did not (“inflamed”). A total of 64 mice were used in the microarry study, in four independent infection experiments. RNA was isolated, amplified, and equal amounts were assayed using Agilent SurePrint G3 Gene Expression 8 × 60 Microarray chips. Scanned data were normalized (80th percentile) and gene expression data analyzed using Genespring v9. Differentially expressed (DE) genes were defined using a false discovery rate (FDR) of 5%. Source data are accessible from EBI Array Express (E-MEXP-3877) and methodology for subsequent data analysis is described in further detail elsewhere (31).

### Histological Analysis

Mice were treated with 1 µg recombinant IL-15 (BioLegend) intravenously and infected 3 days later. Four days postinfection, mice livers were extracted, weighed, and then placed into 2% PFA in PBS for 2 h, then 30% sucrose in PBS overnight. Tissues were then embedded in optimal cutting temperature (Sakura) and stored at −70°C until use. 10 µm cryosections were fixed and labeled with Alexa647 or Alexa488 conjugated F4/80 (eBioscience) and DAPI (Invitrogen) to visualize KCs and cell nuclei, respectively. Images were captured as 0.81 µm optical slices using a LSM510 confocal microscope (Zeiss). Blinded slides were imaged to score the percentage of infected foci having formed a distinct inflammatory focus (greater than 15 cells), with imaging fields selected *via* tdTomato expression.

### Flow Cytometry

Livers were homogenized and mononuclear cells prepared as previously described (30). Cells were incubated with anti-CD16/32 and then labeled with NK1.1, CD3, B220, and CD1d tetramer (a kind gift from V. Cerundulo) to identify T, NK, and NKT cells. Samples were analyzed using a CyAn flow cytometer with Summit software (DAKO). Autofluorescent events and dead cells were excluded from analysis by gating on unused fluorescent channels and LIVE/DEAD fixable dead cell stain (Invitrogen), respectively.

### Parameterizing and Calibrating the Simulation

A full summary of the biological data available that was used to calibrate the simulation is listed in Table S1 in Supplementary Material. The entire list of baseline simulation parameters is found in Table S3 in Supplementary Material. Full details of parameterization and calibration of the simulation are provided in the Supplemental Experimental Procedures.

### Statistical Analysis

When quantifying granulomas, experimental data are expressed as mean ± SEM for each group of five mice from two independent experiments, and statistical analyses performed using two-tailed paired Student’s *t*-tests. All tests used 95% confidence intervals. Simulation data non-normality was determined using the D’Agostino and Pearson test, and non-normal simulation data were analyzed using either Wilcoxon signed-rank or Kolmogorov–Smirnov tests where appropriate. Aleatory analysis was used to determine the minimum number of simulation results required to mitigate stochastic uncertainty (see Figure S4 in Supplementary Material). Latin-hypercube sensitivity analysis was facilitated by using the Spartan tool for understanding uncertainty in simulations (32).

## Results

### Chemokine Production by KCs in Mice Infected With *L. donovani*

**Figure 1:**
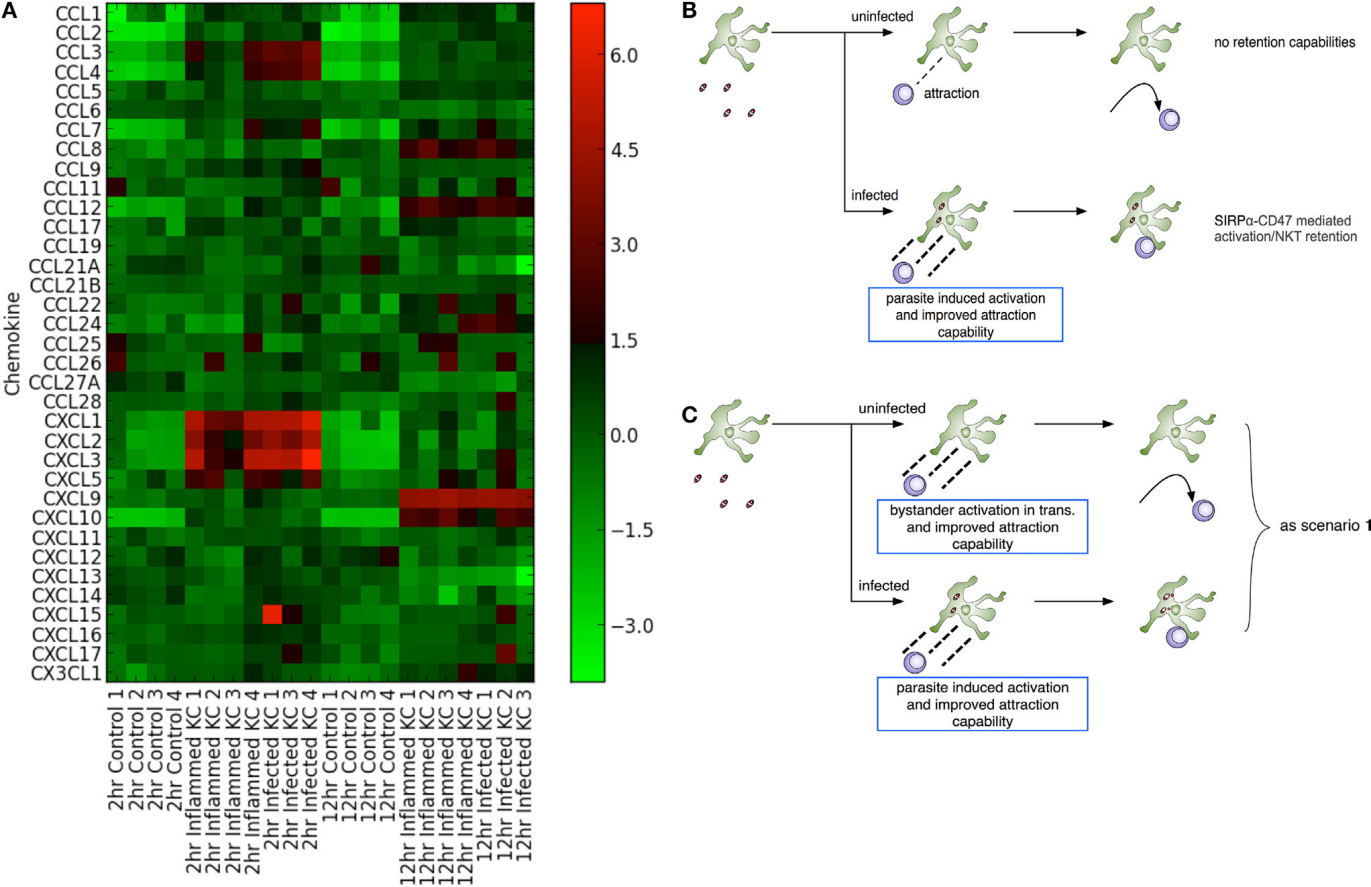
*Leishmania donovani* infection induces transactivation of Kupffer cells (KCs) for chemokine production. **(A)** Heat map showing chemokine mRNA abundance in flow sorted KCs from naïve mice (control) and from KCs isolated from infected mice and separated into those containing parasites (“infected”) and those that do not contain parasites (“inflamed”). KC isolation was performed at 2 and 12 h postinfection, with matched controls. Lanes numbered 1–4 indicates separate sorts. The gating strategy for separating “infected” from “inflamed” KCs is provided in Figure 3 of Ref. (31). **(B,C)** Two modeling scenarios were generated. In scenario 1 **(B)**, only infected KCs produce sufficient chemokine to attract and retain natural killer T (NKT) cells. In scenario 2 **(C)**, both infected and inflamed KCs produce chemokines to attract NKT cells, although only infected KCs have the ability to retain these through cognate interactions.

Both chemokines and iNKT cells are central to the initiation of granulomatous inflammation following *L. donovani* infection. In order to gain insight into the production of chemokines involved in KC-directed recruitment of NKT cells, we used transcriptional profiling of KCs isolated from mice infected with *L. donovani* as previously described (31). Following infection of mice with Td-tomato transgenic *L. donovani*, approx. 20% of the KC population are infected with amastigotes. We isolated KCs from infected mice and sort purified these KCs on the basis of whether they contained intracellular amastigotes (“infected”) or not (herein referred to as “inflamed” to denote their exposure to inflammatory signals *in vivo*) (31). As shown in Figure 1A, KCs from infected mice expressed a variety of chemokines when compared to KCs isolated from naïve mice. At 2 h post infection (p.i.), enhanced accumulation of mRNAs for *Cxcl1, Cxcl2, Cxcl3*, and *Cxcl5*, as well as *Ccl3* and *Ccl4*, was evident (determined as differentially expressed using a 5% FDR). This transcriptional response was transient, in keeping with previous studies at the level of whole liver tissue (21). Rapid secretion of chemokines in response to *L. donovani* infection can also be inferred from studies in which G-protein coupled receptor signaling was abrogated by pertussis toxin (22). A suite of inducible chemokines, including *Cxcl9, Cxcl10, Ccl8*, and *Ccl12* showed enhanced mRNA accumulation at 12 h p.i. (at a 5% FDR), again in keeping with data in whole liver and with previously published data indicating the production of IFNγ by iNKT cells during early *L. donovani* infection [e.g., Figure 2 in Ref. (22)]. For example, qRT-PCR demonstrated sustained and elevated *Cxcl10* at 24 h p.i. (33). Similarly, transcriptional profiling of the livers of infected BALB/c mice (*n* = 4–5 per time point) indicates sustained elevation of *Cxcl9* (Log_2_FC compared to controls of 5.25, 5.14, 5.34, and 4.74 for days 15, 21, 36, and 42 p.i., respectively; FDR 0.05, *p* < 0.05) and *Cxcl10* (Log_2_FC of 4.84, 4.92, 5.36, and 4.55, respectively; Ashwin et al., manuscript in preparation). Strikingly, there was little difference to discriminate the chemokine response of infected vs. inflamed KCs, although we cannot rule out different degrees of posttranscriptional regulation of chemokine secretion in infected vs. inflamed KCs (34). Collectively, our data suggest that although initiated by infection, production of chemokines rapidly spreads in trans throughout the liver KC network.

Chemokine induction by infected cells is thought to provide a means for focal inflammation, the recruitment of additional leukocytes in an ordered manner being essential for granuloma formation and the ultimate activation of macrophage host defense mechanisms. However, given this argument, these data appear counterintuitive. In order to try to understand how transactivation for chemokine production might influence the generation of focal inflammation, and given the absence of tools to selectively and directly manipulate chemokine production by infected vs. uninfected KCs *in vivo*, we adopted an *in silico* experimental approach conducive to testing a variety of different hypotheses (Figures 1B,C).

### An ABM of the Hepatic Sinusoidal Microenvironment

**Figure 2:**
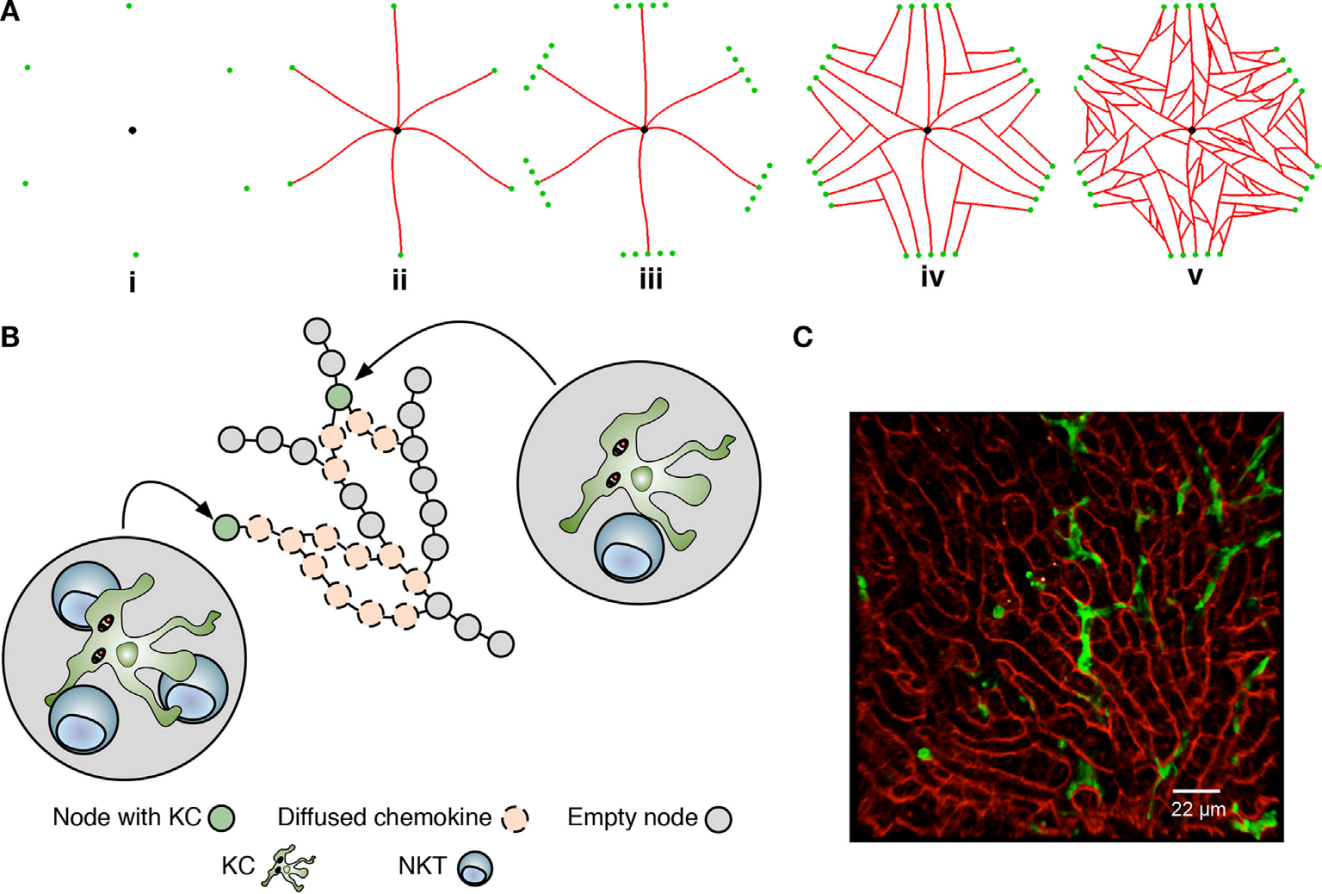
Overview of the liver agent-based model. **(A)** A simulated sinusoidal network was constructed in quasi-2D space using a sinusoidal structure generation algorithm (see Supplemental Experimental Procedures). A drain node representing the portal vein (black) is placed in the center of a 2D space with six surrounding entry nodes representing the portal triads (green), forming an irregular hexagon layout (i). Sinusoids (red) are grown from entry nodes to the drain node (ii). Additional entry nodes created around original entry nodes conceptual form a portal triad (iii), allow additional sinusoids to be grown (iv). Additional sinusoid branches are added between existing sinusoids (v). Execution of the algorithm is shown in Movie S1 in Supplementary Material. **(B)** Node structure of the model underlying KC placement and chemokine diffusion. Nodes are populated or not with a single KC and may attract natural killer T cells to that node. Chemokines exert their effect by “diffusing” across nodes. For further details, see text and Supplementary Experimental Procedures. **(C)** Snapshot of 2-photon image of liver from (mT/mG × lysMcre)_F1_ mice, showing sinusoids (red) and KCs (green).

Agent-based models, where rule-driven “agents” can represent a cell or lower-scale entities of interest, are naturally suited to simulating inflammation in a spatially constrained environment (35–37). To construct this environment, we used published 3D data describing the overall size of lobules, the average non-branched sinusoid length, and the branching angles between sinusoids (38) as the basis for developing a novel algorithm to generate statistically realistic liver lobule sections similar to that reported recently (39). A range of quasi-2D sinusoidal network structures, where each structure can be considered as a slice through a 3D lobule, was created using a multi-stage generative algorithm augmented with these data (38) (Figure 2A; Movie S1, Figures S1A–D, Table S1, and Supplementary Experimental Procedures in Supplementary Material). The resulting networks (Figure 2B), represented as graphs of nodes connected by edges, serve as discrete spatial simulation environments that mimic the sinusoidal structure observed in live mice imaged by 2-photon intra-vital microscopy in (mT/mG × lysMcre)_F1_, as previously described (30) (Figure 2A vs Figure 2C). Analysis (by Pearson correlation coefficients and Kolmogorov–Smirnov tests) using 10 independently generated structures indicated that variance in structure *per se* had minimal impact on the results of subsequent simulations (see below).

We defined where and how cellular interactions were allowed to occur within our simulation environment based on three different types of network node: periportal nodes, located at the peripheries of the structure allow NKT cells to enter and exit the simulated lobule section; regular-nodes, capable of holding a single KC and any number of NKT cells; and a single centrilobular-node, representing the central vein where NKT cells could exit the structure. Only NKT cells were capable of movement within the structure. KCs remain immobile, as reported in early stages of infection with *B. bugdorferi* (25), BCG (40), and *L. donovani* (30). Our KC placement algorithm distributes KCs in periportal, midzonal, and centrilobular locations in a ratio of 4:3:3, based on Ref. (41, 42). As centrilobular KCs have reduced phagocytic capability compared to periportal KCs (41), the distribution of infected KCs in our simulation is 65% periportal, 25% midzonal, and 10% centrilobular for the purposes of experimentation.

A detailed description of the model and key assumptions is provided in the Supplemental Experimental Procedures and Tables S2 and S3 in Supplementary Material. State diagrams written in the Unified Modeling Language that illustrate the behavior associated with KCs and NKT cells are provided in Figure S2 in Supplementary Material. Briefly, mechanisms of cellular attraction and retention were modeled generically, since the precise function, functional overlap, and interaction between distinct chemokines has yet to be fully elucidated. For the purposes of the current abstraction, we refer to the chemokines as attractive and retentive, being independent and quantitatively distinct and with discrete areas of influence. The simulation was constructed to allow both a minimum and a maximum diffusion distance to be parameterized for all chemo-attractants produced by KCs. NKT cells traverse the sinusoidal network at 10–20 µm/min with a random walk behavior (25), with no enforcement of directionality unless under the attractive influence of KC-derived chemokines. Strength of attraction is modeled as a function of distance from the source KC. Upon interaction with infected KCs, NKT cells produce IFNγ (as a representation of all macrophage-activating cytokines) following cognate receptor engagement (22), facilitating KC activation and NKT cell arrest (25, 43). Our previous data on SIRPα-CD47 have suggested that cognate receptor–ligand interactions also regulate NKT cell retention on infected KCs, with the induced expression of SIRPα after infection being preferentially, but not exclusively observed on infected KCs (22). In our model, this interaction is used to represent a cognate retention signal, but this reflects an abstraction of what may be potentially much more complex interactions. The amplification of KC-derived attractive chemokines through this process can lead to the accumulation of multiple NKT cells at a given KC (referred to here as “inflammatory foci”). It is assumed that through the sum of all KC–NKT cell interactions within an inflammatory focus, a threshold for granuloma formation and the subsequent recruitment of additional leukocytes associated with maturing granulomas (including B cells, T cells, monocytes, and NK cells) is reached, but these cells and processes are not explicitly modeled. We have also not modeled the ultimate microbicidal activity of these granulomas.

### Parasite-Induced Activation of Infected KCs With/Without Bystander Chemokine Production by Uninfected KCs

Two experimental scenarios were devised to investigate the influence of varying both infected and inflamed KC function. Scenario 1 (Figure 1B) was constructed to restrict chemokine production to infected KCs only, and scenario 2 (Figure 1C) to investigate the impact of transactivation of KC for chemokine production. As KC activation of NKT cells is optimal in the presence of cognate interactions (22), our model assumes these are a requirement for retention; hence, only infected KCs can generate stable inflammatory foci, and these foci, for the purposes of the model, are composed only of NKT cells and KCs. In contrast, inflamed KCs in scenario 2 might act as potential competitors for available NKT cells, being able to attract but not retain them. Although this model can be used to probe a variety of different potential questions related to the initiation of granuloma formation (see Discussion), we focus here on a factorial analysis that involved simultaneously modifying the simulation parameters related to chemokine diffusion distance, time required to activate KCs, and time for KCs to reach maximal chemokine production.

First, we quantified the influence of distance from effect on attraction. Factorial analysis, modifying the maximum diffusion distance of chemokine, showed that greater chemokine diffusion distance leads to increased percentages of infected KCs forming inflammatory foci in both scenarios (Figures 1B,C), whether those foci were qualified as containing 4, 6, or 8 NKT cells. However, our simulation predicted diminishing returns when increasing maximum diffusion past ~120 μm (Figure S1E in Supplementary Material). Thus, significant differences (*P* ≤ 0.001) were observed when comparing the frequency of inflammatory foci that resulted from each increase in diffusion distance against the previous distance (e.g., 20–30 μm: *P* = 0.001216, 30–40 μm: *P* = 0.000019). However, when increasing from 120 to 130 µm and beyond, the increase in inflammatory foci was not significant (*P* = 0.312). Interestingly, this tipping point is close to the ~100 μm reported as the distance of a functional chemokine gradient *in vivo* (44). These results suggest that if it were possible to selectively increase chemokine diffusion *via* increased production (or other means) by infected KCs compared to inflamed KCs, or conversely decrease chemokine diffusion by inflamed KCs, infected KCs would gain competitive advantage in terms of attracting NKT cells.

**Figure 3:**
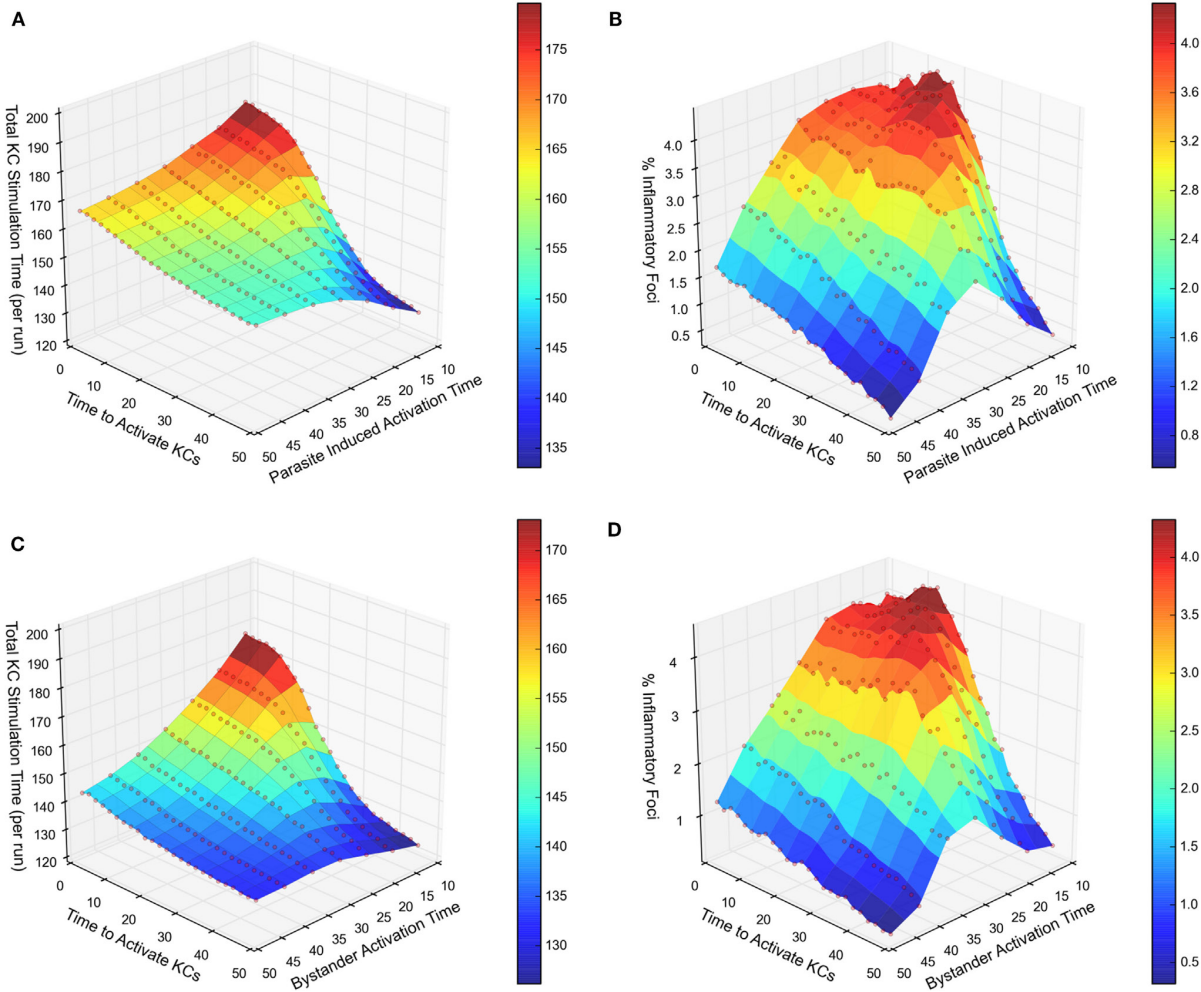
Response landscapes for parasite-induced KC activation with and without KC activation in trans. **(A–D)** Two-at-a-time (TAT) parameter analysis showing the effect on total KC stimulation time **(A,C)** and on % inflammatory foci **(B,D)** of modifying either cumulative time to activate KCs and parasite-induced activation time **(A,B)** or cumulative time to activate KCs and bystander activation time **(C,D)**. For further details, see Supplementary Experimental Procedures.

We next compared our two experimental scenarios in terms of total stimulation time (i.e., a measure of activation) received by the entire infected KC population, and the frequency of inflammatory foci formed associated with that population. Figure 3A illustrates a response curve for scenario 1 showing the total stimulation time received by all infected KCs, across a range of the two main parameters that determine KC activation dynamics—the time required to activate KCs and the duration KCs remain activated. When comparing this response landscape of scenario 1 with that generated in scenario 2 (Figure 3C), there was a marked reduction in stimulation time received overall by infected KCs in scenario 2 compared to scenario 1. This trend is also observable when comparing the percentage of inflammatory foci, whether qualified at 8 NKT cells (Figure 3B for scenario 1 and Figure 3D for scenario 2) or at 4 or 6 NKT cells (data not shown).

Together, these results demonstrate that in comparison to chemokine production restricted to infected KCs, additional chemokine production by inflamed KC generates a less focused inflammatory response, measured either by frequency of infected KC that form inflammatory foci, or by stimulation time received by infected KCs. This result most likely reflects the liver lobule becoming saturated with attractive chemokines derived from both inflamed and infected KCs in scenario 2, reducing the competitiveness of infected KCs to selectively recruit NKT cells. In other words, chemokine production by inflamed KC acts in a negative immune regulatory manner, limiting the extent of the inflammatory response around infected KCs.

### Increasing NKT Cell Numbers Overcomes Bystander Regulation

**Figure 4:**
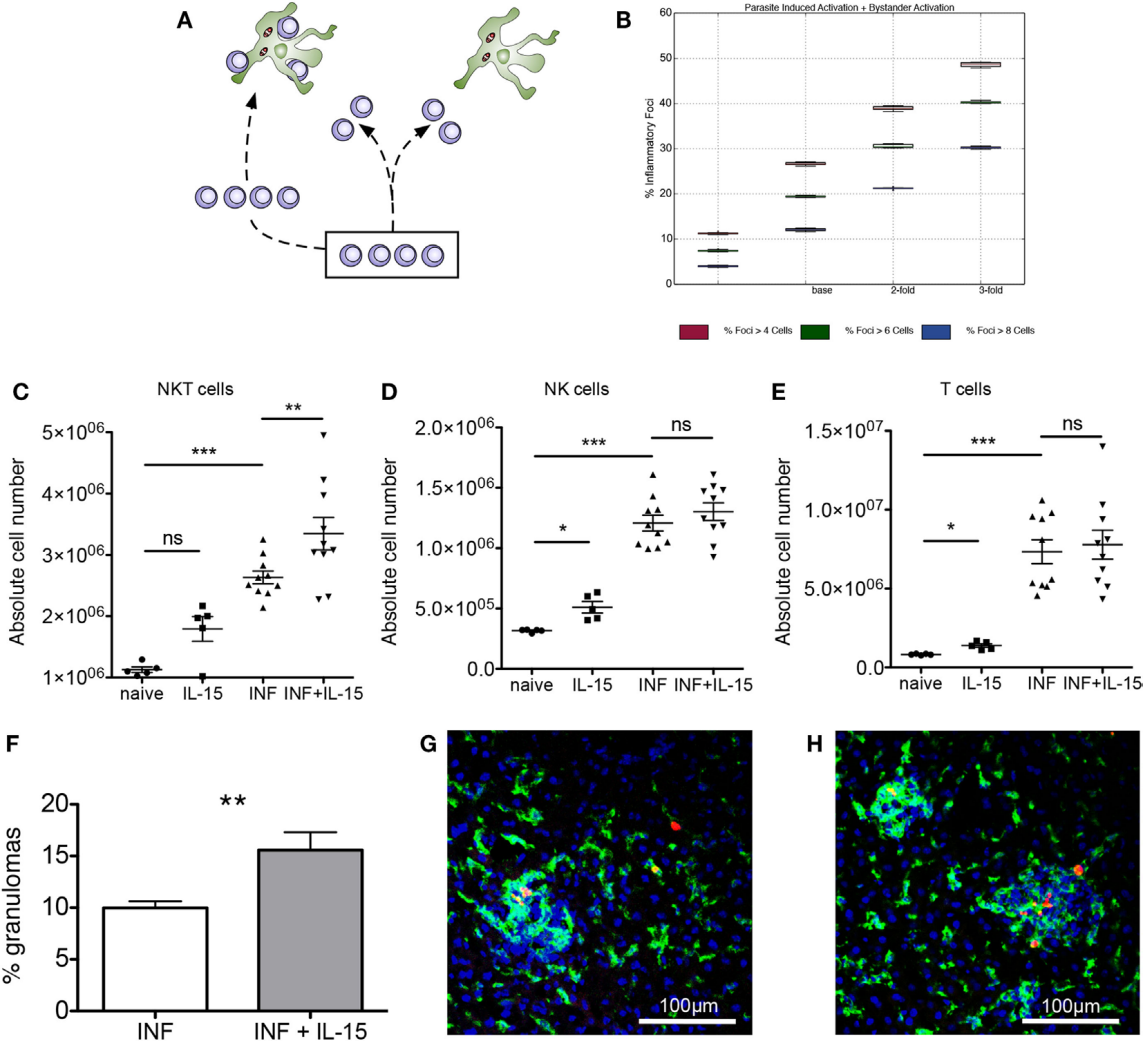
Expansion of natural killer T (NKT) cells promotes granuloma formation. **(A)** Alternate hypotheses for impact of increasing NKT cell number. **(B)** Increasing NKT cells *in silico* leads to greater percentages of KCs that form an inflammatory focus, when qualified at 4, 6, and 8 cells. **(C–E)** Absolute numbers of NKT **(C)**, NK **(D)**, and T cells **(E)** in naïve and infected mice with or without administration of rIL-15. Results are pooled from two independent experiments and represent mean ± SEM (*n* = 10 mice per group). *P < 0.05, **P < 0.01, ***P < 0.001, by paired Student’s *t*-test. **(F)** Percentage of infected KCs with surrounding granuloma in control and rIL-15-treated infected mice. **P < 0.01 (*n* = 10 mice). **(G,H)** Heterogeneity of granulomas comparing infected **(G)** and rIL-15-treated **(H)** mice infected with TdTomato-*Leishmania donovani* (red). Sections were stained using F4/80 (green) and counterstained with DAPI (blue).

We then investigated how modifying the target of this competition affected the quantity and quality of inflammatory foci generated. We hypothesized that altering NKT cell frequency might result in either (i) similarly abundant foci, but with each being more substantive in terms of NKT cellularity or (ii) increased numbers of inflammatory foci, thus overcoming the competitive effect of bystander chemokine production by inflamed KCs (Figure 4A). Our simulation results showed that increasing NKT cell numbers above the calibrated value lead to significant increases in the frequency of inflammatory foci in scenario 1, a result that would be expected. Strikingly, an increase in frequency of inflammatory foci was also observed to be the case for scenario 2, regardless of how we qualified focus size (Figure 4B). For example, with an increase in NKT cell availability of twofold, the number of inflammatory foci increased 1.5-fold, whereas increasing NKT cells by threefold doubled the frequency of inflammatory foci.

To test whether this predictive *in silico* data was also borne out *in vivo*, we treated mice for 3 days with recombinant IL-15 to induce increased NKT cell proliferation and survival (45) and then infected these mice with *L. donovani* and scored early granuloma formation. In uninfected mice, IL-15 treatment resulted in increased numbers of NKT cells (including CD1d restricted NKT cells), NK cells, and T cells (Figures 4C–E; Figures S3A–D in Supplementary Material). In infected mice, all cell types were already increased in number compared to naïve mice, and the effect of IL-15 pretreatment was limited to an increase in the number of NKT cells (Figure 4C). Similarly, IL-15 pretreatment had no effect on the relative frequency of NK cells and T cells (Figures S3B,C in Supplementary Material) but resulted in an increase in the relative frequency of NKT cells (from 15.0 ± 0.1 to 17.36 ± 0.8%; *n* = 10; *P* = 0.0043; Figure S3E in Supplementary Material).

To ensure that we were scoring a biologically relevant histopathological response, while minimizing potential longer terms effects of rIL-15 treatment, we chose to score the granulomas early in their development (day 4 p.i.) and define these as accumulations of 15 or more cells formed around an infected KC (not discriminating between NKT cells or other mononuclear cells). Although there was significant heterogeneity in size of these granulomas (Figures 4G,H), we found that the frequency of infected KCs that formed distinct granulomas was increased ~1.5-fold in mice pretreated with IL-15 and which had a higher number of NKT cells in the liver at the time of infection (*P* = 0.0038; Figure 4F). Thus, treatment of mice with rIL-15, even under conditions where the increase in NKT cell number is relatively modest, leads to a significant enhancement in the frequency of infected KCs that can provide a nidus for granuloma formation.

## Discussion

Granulomas represent a specialized form of inflammation that allows for the focal delivery of host effector responses and/or containment of pathogen products. While generally considered host beneficial, excessive granuloma formation may have significant pathological consequences. Here, we provide evidence that chemokine-dependent competition between infected and uninfected KCs for iNKT cells in the hepatic microenvironment acts as a natural attenuator of granuloma formation.

In models of experimental visceral leishmaniasis, granuloma formation is asynchronous, limiting the extent of hepatic inflammation, but also delaying parasite clearance (16, 27). A variety of different models could explain why isolated infected KCs can be found at times when other infected KCs are engaged in a fully mature granulomatous response. In a model of *Mycobacterium marinarum*-induced granulomas in zebrafish, macrophage migration out of the granuloma has been observed (14, 46), and it is possible that infected KCs leave granulomas in mice infected with *L. donovani*. However, in both *L. donovani*-induced granulomas (30) and BCG-induced granulomas (40, 47) in immunocompetent mice, KCs appear to retain their characteristic lack of motility. Alternatively, there may be heterogeneity in KCs, a subset being more efficient in promoting granulomatous inflammation. Although we had previously modeled this possibility using an early version of our ABM (48), our recent studies evaluating differences between yolk-sac derived and bone marrow-derived KC indicate that both are competent to form granulomas and participate effectively in this response (49). A further possibility is that granuloma formation is rate limited by the availability of key amplifier cells. Experimental data to date indicate that iNKT cells play this role in experimental visceral leishmaniasis (22, 33, 50, 51), though we do not discount a role for other more recently identified innate lymphoid cells (52, 53).

Through transcriptional profiling, we demonstrated that both inflamed and infected KCs produce a variety of inducible chemokines able to attract NKT cells, suggesting the possibility that uninfected as well as infected KCs could compete for this resource. However, as neither the mechanisms that regulate this transactivation nor experimental means to selectively regulate chemokine production by KCs are currently available, we adopted a computational approach to further explore this hypothesis. ABMs are well-suited toward studying tissue and cellular level inflammation (35–37). In constructing our ABM, we developed a novel algorithm for creating virtual sinusoidal networks that are visually representative of liver lobule sections, being defined by published statistics that captured the length between central vein and portal triad, average lengths of non-branched sinusoids and sinusoid branch angles (38). This represents an improvement on similar work (39). Our algorithm was not intended to produce a fully realistic whole lobule structure, but rather we were interested only in developing suitable quasi-2D vascular networks within liver lobules to provide an environment for the cellular and chemokine “agents” contained in the model. Similarly, while our ABM contained only three cellular agents (infected and inflamed KCs and NKT), this abstraction was nevertheless sufficient to probe previously inaccessible aspects of the underlying biology.

Our *in silico* results predicted that chemokine diffusion plays an important role in regulating the formation of inflammatory foci around infected KCs, though there are diminishing returns as a result of increased competition when lobules become flooded with chemokines. Subsequently, our model predicted an intuitive, but nonetheless previously unreported mechanism by which the production of NKT cell-attractive chemokines by inflamed KCs dampens the overall inflammatory response in the liver microenvironment, reducing the activation received by infected KCs. Our *in silico* data also predicted that this competition could be overcome by increasing the availability of NKT cells, and we were able to confirm that granuloma frequency can indeed be increased *in vivo* by increasing NKT cell numbers using rIL-15. The relationship between availability of NKT cells and an increase in the frequency of infected KCs generating granulomas has not previously been demonstrated.

Natural killer T cells represent a potent therapeutic target in a variety of clinical settings, due to their immune adjuvant function and production of various effector cytokines (54–57). Protective immunity associated with NKT cell activation has been reported in several disease settings. For example, Vα14 NKT cells activated by α-galactosylceramide (α-GalCer) have been shown to inhibit the development of malaria parasites in mice (58). Similarly, in a murine model of *Mycobacterium tuberculosis* infection, α-GalCer-induced activation of NKT cells was associated with reduced bacterial loads, tissue injury, and improved mouse survival (59). Conversely, NKT cells have been implicated as key drivers of liver inflammation such as chronic liver injury (26). Although our results suggest that, in leishmaniasis, the initiation of granulomatous inflammation can be enhanced by increasing the availability of NKT cells, further long-term studies would be required to determine whether the host protective advantages of this intervention outweigh any possible pathological consequences.

It is important to recognize that our model has been developed to address early events in granuloma formation and does not take into account the potential for diversity in granuloma form and function, including variations in microbicidal activity. These may be regulated *via* other aspects of the immune response that develop over time, and more complex models have been developed to address some of these issues (60). Redundancy of immune regulatory pathways is a common finding, and it is possible that other mechanisms come into play at later stages of granuloma evolution that affects the ability of KCs to recruit inflammatory cells and initiate granuloma formation. The kinetics of chemokine production is also likely to be highly dynamic, though in respect of CXCL9 and CXCL10, long-term transcriptomic profiling indicates that expression of these IFNγ-inducible chemokines is sustained for at least 45 days postinfection (Ashwin et. al., unpublished).

In summary, our data argue that chemokine production by uninfected transactivated KCs provides an example of a novel negative regulatory mechanism to limit the impact of overzealous inflammatory responses that might otherwise lead to excess tissue pathology. Further studies to evaluate this hypothesis in a broader context of inflammation are clearly warranted.

## Ethics Statement

This study was carried out in compliance with the Animals (Scientific Procedures) Act 1986, under UK Home Office license PPL 60/4377. The protocol was approved by the University of York Animal Welfare and Ethical Review Board.

## Author Contributions

DM, PA, JT, LB, and PK designed the simulation model. DM implemented the simulation model. LB and PK designed the experimental study. DM, JM, LB, and AS performed experimental studies. SH provided data and input on model development. PA and AS designed and implemented the algorithm for the generation of the artificial sinusoid structures. DM, LB, PA, JT, and PK analyzed the data and wrote the manuscript.

## Conflict of Interest Statement

JT is Director of SimOmics Ltd.; PA is employed by SimOmics Ltd.; all other authors declare no conflict of interest.

## References

[1] Isaacs A Lindenmann J Virus interference. I. The interferon. Proc R Soc Lond B Biol Sci (1957) 147(927):258–67. 10.1098/rspb.1957.0048 13465720

[2] Isaacs A Lindenmann J Valentine RC Virus interference. II. Some properties of interferon. Proc R Soc Lond B Biol Sci (1957) 147(927):268–73. 10.1098/rspb.1957.0049 13465721

[3] Mackaness GB. The influence of immunologically committed lymphoid cells on macrophage activity in vivo. J Exp Med (1969) 129(5):973–92. 10.1084/jem.129.5.973 4976110 PMC2138649

[4] Chu T Tyznik AJ Roepke S Berkley AM Woodward-Davis A Pattacini L Bystander-activated memory CD8 T cells control early pathogen load in an innate-like, NKG2D-dependent manner. Cell Rep (2013) 3(3):701–8. 10.1016/j.celrep.2013.02.020 23523350 PMC3628815

[5] Griffith JW Sokol CL Luster AD. Chemokines and chemokine receptors: positioning cells for host defense and immunity. Annu Rev Immunol (2014) 32:659–702. 10.1146/annurev-immunol-032713-120145 24655300

[6] Lertmemongkolchai G Cai G Hunter CA Bancroft GJ. Bystander activation of CD8+ T cells contributes to the rapid production of IFN-gamma in response to bacterial pathogens. J Immunol (2001) 166(2):1097–105. 10.4049/jimmunol.166.2.1097 11145690

[7] Mantovani A Sica A Sozzani S Allavena P Vecchi A Locati M. The chemokine system in diverse forms of macrophage activation and polarization. Trends Immunol (2004) 25(12):677–86. 10.1016/j.it.2004.09.015 15530839

[8] Polley R Sanos SL Prickett S Haque A Kaye PM. Chronic *Leishmania donovani* infection promotes bystander CD8+-T-cell expansion and heterologous immunity. Infect Immun (2005) 73(12):7996–8001. 10.1128/IAI.73.12.7996-8001.2005 16299292 PMC1307086

[9] Tough DF Borrow P Sprent J. Induction of bystander T cell proliferation by viruses and type I interferon in vivo. Science (1996) 272(5270):1947–50. 10.1126/science.272.5270.1947 8658169

[10] Graham AL Allen JE Read AF Evolutionary causes and consequences of immunopathology. Ann Rev Ecol Evol Syst (2005) 36:373–97. 10.1146/annurev.ecolsys.36.102003.152622

[11] Mills KH. Regulatory T cells: friend or foe in immunity to infection? Nat Rev Immunol (2004) 4(11):841–55. 10.1038/nri1485 15516964

[12] Sorci G Cornet S Faivre B. Immune evasion, immunopathology and the regulation of the immune system. Pathogens (2013) 2(1):71–91. 10.3390/pathogens2010071 25436882 PMC4235712

[13] Dorhoi A Kaufmann SH. Perspectives on host adaptation in response to *Mycobacterium tuberculosis*: modulation of inflammation. Semin Immunol (2014) 26(6):533–42. 10.1016/j.smim.2014.10.002 25453228

[14] Pagan AJ Ramakrishnan L Immunity and immunopathology in the tuberculous granuloma. Cold Spring Harb Perspect Med (2015) 5(9). 10.1101/cshperspect.a018499 PMC4561401 25377142

[15] Hams E Aviello G Fallon PG. The schistosoma granuloma: friend or foe? Front Immunol (2013) 4:89. 10.3389/fimmu.2013.00089 23596444 PMC3625856

[16] Kaye PM Beattie L. Lessons from other diseases: granulomatous inflammation in leishmaniasis. Semin Immunopathol (2016) 38(2):249–60. 10.1007/s00281-015-0548-7 26678994 PMC4779128

[17] Lundy SK Lukacs NW. Chronic schistosome infection leads to modulation of granuloma formation and systemic immune suppression. Front Immunol (2013) 4:39. 10.3389/fimmu.2013.00039 23429492 PMC3576626

[18] Maroof A Beattie L Zubairi S Svensson M Stager S Kaye PM. Posttranscriptional regulation of II10 gene expression allows natural killer cells to express immunoregulatory function. Immunity (2008) 29(2):295–305. 10.1016/j.immuni.2008.06.012 18701085 PMC2656759

[19] Mentink-Kane MM Cheever AW Thompson RW Hari DM Kabatereine NB Vennervald BJ IL-13 receptor alpha 2 down-modulates granulomatous inflammation and prolongs host survival in schistosomiasis. Proc Natl Acad Sci U S A (2004) 101(2):586–90. 10.1073/pnas.0305064101 14699044 PMC327191

[20] Owens BM Beattie L Moore JW Brown N Mann JL Dalton JE IL-10-producing Th1 cells and disease progression are regulated by distinct CD11c(+) cell populations during visceral leishmaniasis. PLoS Pathog (2012) 8(7):e1002827 10.1371/journal.ppat.1002827 22911108 PMC3406093

[21] Cotterell SE Engwerda CR Kaye PM. *Leishmania donovani* infection initiates T cell-independent chemokine responses, which are subsequently amplified in a T cell-dependent manner. Eur J Immunol (1999) 29(1):203–14. 10.1002/(SICI)1521-4141(199901)29:01<203::AID-IMMU203>3.0.CO;2-B 9933102

[22] Beattie L Svensson M Bune A Brown N Maroof A Zubairi S *Leishmania donovani*-induced expression of signal regulatory protein alpha on Kupffer cells enhances hepatic invariant NKT-cell activation. Eur J Immunol (2010) 40(1):117–23. 10.1002/eji.200939863 19877019 PMC2909397

[23] Gupta G Bhattacharjee S Bhattacharyya S Bhattacharya P Adhikari A Mukherjee A CXC chemokine-mediated protection against visceral leishmaniasis: involvement of the proinflammatory response. J Infect Dis (2009) 200(8):1300–10. 10.1086/605895 19743920

[24] Sato T Thorlacius H Johnston B Staton TL Xiang W Littman DR Role for CXCR6 in recruitment of activated CD8+ lymphocytes to inflamed liver. J Immunol (2005) 174(1):277–83. 10.4049/jimmunol.174.1.277 15611250

[25] Lee WY Moriarty TJ Wong CH Zhou H Strieter RM van Rooijen N An intravascular immune response to *Borrelia burgdorferi* involves Kupffer cells and iNKT cells. Nat Immunol (2010) 11(4):295–302. 10.1038/ni.1855 20228796 PMC5114121

[26] Wehr A Baeck C Heymann F Niemietz PM Hammerich L Martin C Chemokine receptor CXCR6-dependent hepatic NK T Cell accumulation promotes inflammation and liver fibrosis. J Immunol (2013) 190(10):5226–36. 10.4049/jimmunol.1202909 23596313

[27] Murray HW. Tissue granuloma structure-function in experimental visceral leishmaniasis. Int J Exp Pathol (2001) 82(5):249–67. 10.1046/j.1365-2613.2001.00199.x 11703536 PMC2517779

[28] Muzumdar MD Tasic B Miyamichi K Li L Luo L A global double-fluorescent Cre reporter mouse. Genesis (2007) 45(9):593–605. 10.1002/dvg.20335 17868096

[29] Clausen BE Burkhardt C Reith W Renkawitz R Forster I. Conditional gene targeting in macrophages and granulocytes using LysMcre mice. Transgenic Res (1999) 8(4):265–77. 10.1023/A:1008942828960 10621974

[30] Beattie L Peltan A Maroof A Kirby A Brown N Coles M Dynamic imaging of experimental *Leishmania donovani*-induced hepatic granulomas detects Kupffer cell-restricted antigen presentation to antigen-specific CD8 T cells. PLoS Pathog (2010) 6(3):e1000805. 10.1371/journal.ppat.1000805 20300603 PMC2837408

[31] Beattie L d’El-Rei Hermida M Moore JW Maroof A Brown N Lagos D A transcriptomic network identified in uninfected macrophages responding to inflammation controls intracellular pathogen survival. Cell Host Microbe (2013) 14(3):357–68. 10.1016/j.chom.2013.08.004 24034621 PMC4180915

[32] Alden K Read M Timmis J Andrews PS Veiga-Fernandes H Coles M. Spartan: a comprehensive tool for understanding uncertainty in simulations of biological systems. PLoS Comput Biol (2013) 9(2):e1002916. 10.1371/journal.pcbi.1002916 23468606 PMC3585389

[33] Svensson M Zubairi S Maroof A Kazi F Taniguchi M Kaye PM. Invariant NKT cells are essential for the regulation of hepatic CXCL10 gene expression during *Leishmania donovani* infection. Infect Immun (2005) 73(11):7541–7. 10.1128/IAI.73.11.7541-7547.2005 16239557 PMC1273891

[34] Fan J Heller NM Gorospe M Atasoy U Stellato C. The role of post-transcriptional regulation in chemokine gene expression in inflammation and allergy. Eur Respir J (2005) 26(5):933–47. 10.1183/09031936.05.00120204 16264057

[35] An G. Concepts for developing a collaborative in silico model of the acute inflammatory response using agent-based modeling. J Crit Care (2006) 21(1):105–10; discussion 110–1. 10.1016/j.jcrc.2005.11.012 16616634

[36] An G Christley S. Addressing the translational dilemma: dynamic knowledge representation of inflammation using agent-based modeling. Crit Rev Biomed Eng (2012) 40(4):323–40. 10.1615/CritRevBiomedEng.v40.i4.70 23140123

[37] Moore JW Moyo D Beattie L Andrews PS Timmis J Kaye PM. Functional complexity of the *Leishmania granuloma* and the potential of in silico modeling. Front Immunol (2013) 4:35. 10.3389/fimmu.2013.00035 23423646 PMC3573688

[38] Hoehme S Brulport M Bauer A Bedawy E Schormann W Hermes M Prediction and validation of cell alignment along microvessels as order principle to restore tissue architecture in liver regeneration. Proc Natl Acad Sci U S A (2010) 107(23):10371–6. 10.1073/pnas.0909374107 20484673 PMC2890786

[39] Wambaugh J Shah I. Simulating microdosimetry in a virtual hepatic lobule. PLoS Comput Biol (2010) 6(4):e1000756. 10.1371/journal.pcbi.1000756 20421935 PMC2858695

[40] Egen JG Rothfuchs AG Feng CG Winter N Sher A Germain RN. Macrophage and T cell dynamics during the development and disintegration of mycobacterial granulomas. Immunity (2008) 28(2):271–84. 10.1016/j.immuni.2007.12.010 18261937 PMC2390753

[41] Bouwens L Baekeland M De Zanger R Wisse E. Quantitation, tissue distribution and proliferation kinetics of Kupffer cells in normal rat liver. Hepatology (1986) 6(4):718–22. 10.1002/hep.1840060430 3733004

[42] Sleyster EC Knook DL. Relation between localization and function of rat liver Kupffer cells. Lab Invest (1982) 47(5):484–90. 6182391

[43] Geissmann F Cameron TO Sidobre S Manlongat N Kronenberg M Briskin MJ Intravascular immune surveillance by CXCR6+ NKT cells patrolling liver sinusoids. PLoS Biol (2005) 3(4):e113. 10.1371/journal.pbio.0030113 15799695 PMC1073691

[44] Weber M Hauschild R Schwarz J Moussion C de Vries I Legler DF Interstitial dendritic cell guidance by haptotactic chemokine gradients. Science (2013) 339(6117):328–32. 10.1126/science.1228456 23329049

[45] Matsuda JL Gapin L Sidobre S Kieper WC Tan JT Ceredig R Homeostasis of V alpha 14i NKT cells. Nat Immunol (2002) 3(10):966–74. 10.1038/ni837 12244311

[46] Davis JM Ramakrishnan L. The role of the granuloma in expansion and dissemination of early tuberculous infection. Cell (2009) 136(1):37–49. 10.1016/j.cell.2008.11.014 19135887 PMC3134310

[47] Egen JG Rothfuchs AG Feng CG Horwitz MA Sher A Germain RN. Intravital imaging reveals limited antigen presentation and T cell effector function in mycobacterial granulomas. Immunity (2011) 34(5):807–19. 10.1016/j.immuni.2011.03.022 21596592 PMC3164316

[48] Flugge AJ Timmis J Andrews PS Moore JW Kaye PM Modelling and simulation of granuloma formation in visceral leishmaniasis. Evolutionary Computation, 2009 CEC ’09; IEEE Congress. (2009). p. 3052–9. 10.1109/CEC.2009.4983329

[49] Beattie L Sawtell A Mann J Frame TC Teal B de Labastida Rivera F Bone marrow-derived and resident liver macrophages display unique transcriptomic signatures but similar biological functions. J Hepatol (2016) 65(4):758–68. 10.1016/j.jhep.2016.05.037 27262757 PMC5028381

[50] Amprey JL Im JS Turco SJ Murray HW Illarionov PA Besra GS A subset of liver NK T cells is activated during *Leishmania donovani* infection by CD1d-bound lipophosphoglycan. J Exp Med (2004) 200(7):895–904. 10.1084/jem.20040704 15466622 PMC2213292

[51] Robert-Gangneux F Drogoul AS Rostan O Piquet-Pellorce C Cayon J Lisbonne M Invariant NKT cells drive hepatic cytokinic microenvironment favoring efficient granuloma formation and early control of *Leishmania donovani* infection. PLoS One (2012) 7(3):e33413. 10.1371/journal.pone.0033413 22457760 PMC3310876

[52] Gasteiger G Fan X Dikiy S Lee SY Rudensky AY. Tissue residency of innate lymphoid cells in lymphoid and nonlymphoid organs. Science (2015) 350(6263):981–5. 10.1126/science.aac9593 26472762 PMC4720139

[53] Robinette ML Fuchs A Cortez VS Lee JS Wang Y Durum SK Transcriptional programs define molecular characteristics of innate lymphoid cell classes and subsets. Nat Immunol (2015) 16(3):306–17. 10.1038/ni.3094 25621825 PMC4372143

[54] Juno JA Keynan Y Fowke KR. Invariant NKT cells: regulation and function during viral infection. PLoS Pathog (2012) 8(8):e1002838. 10.1371/journal.ppat.1002838 22916008 PMC3420949

[55] Mattarollo SR West AC Steegh K Duret H Paget C Martin B NKT cell adjuvant-based tumor vaccine for treatment of myc oncogene-driven mouse B-cell lymphoma. Blood (2012) 120(15):3019–29. 10.1182/blood-2012-04-426643 22932803 PMC3557399

[56] Mussai F De Santo C Cerundolo V. Interaction between invariant NKT cells and myeloid-derived suppressor cells in cancer patients: evidence and therapeutic opportunities. J Immunother (2012) 35(6):449–59. 10.1097/CJI.0b013e31825be926 22735803

[57] Pilones KA Aryankalayil J Demaria S. Invariant NKT cells as novel targets for immunotherapy in solid tumors. Clin Dev Immunol (2012) 2012:720803. 10.1155/2012/720803 23118781 PMC3483734

[58] Gonzalez-Aseguinolaza G de Oliveira C Tomaska M Hong S Bruna-Romero O Nakayama T Alpha-galactosylceramide-activated Valpha 14 natural killer T cells mediate protection against murine malaria. Proc Natl Acad Sci U S A (2000) 97(15):8461–6. 10.1073/pnas.97.15.8461 10900007 PMC26970

[59] Chackerian A Alt J Perera V Behar SM. Activation of NKT cells protects mice from tuberculosis. Infect Immun (2002) 70(11):6302–9. 10.1128/IAI.70.11.6302-6309.2002 12379709 PMC130331

[60] Albergante L Timmis J Beattie L Kaye PM. A Petri net model of granulomatous inflammation: implications for IL-10 mediated control of *Leishmania donovani* infection. PLoS Comput Biol (2013) 9(11):e1003334. 10.1371/journal.pcbi.1003334 24363630 PMC3867212

