# Supplementary material for: Macrophage Transactivation for Chemokine Production Identified as a Negative Regulator of Granulomatous Inflammation Using Agent-Based Modeling
Source: Front Immunol. 2018 Mar 27;9:637. doi: 10.3389/fimmu.2018.00637 (PMC5880939; doi:10.3389/fimmu.2018.00637)
Supplement: Supplementary file 1 [file data_sheet_1.PDF]

Supplemental Information

Macrophage transactivation for chemokine production identified as a negative regulator of granulomatous inflammation using agent-based modeling.

Daniel Moyo[1,2], Lynette Beattie[1*], Paul S Andrews[3], John W J Moore[1], Jon Timmis[3], Amy Sawtell[1], Stefan Hoehme[4], Adam T. Sampson[5], Paul M Kaye[1]

Supplemental Figures

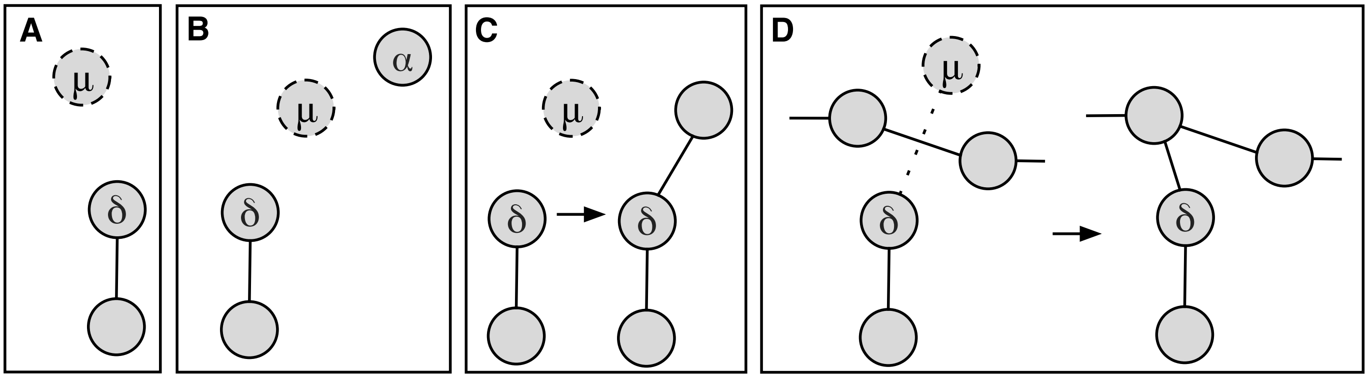

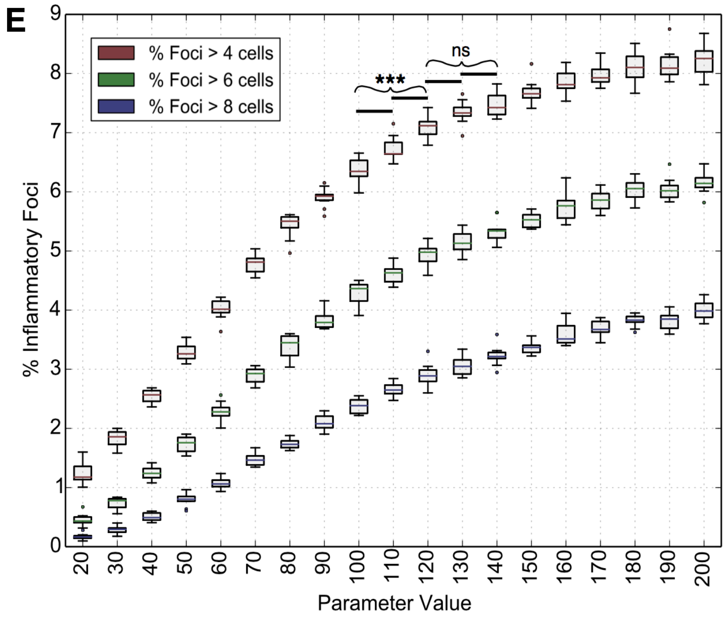

Figure S1. Related to Figure 2. Generation of Nodes and Edges of a Sinusoid Network.
(A-D) See Algorithm 1 in Supplemental Experimental Procedures. (E) Effect on percentage inflammatory foci qualified at 4, 6 and 8 cells, when modifying maximum chemokine diffusion distance.

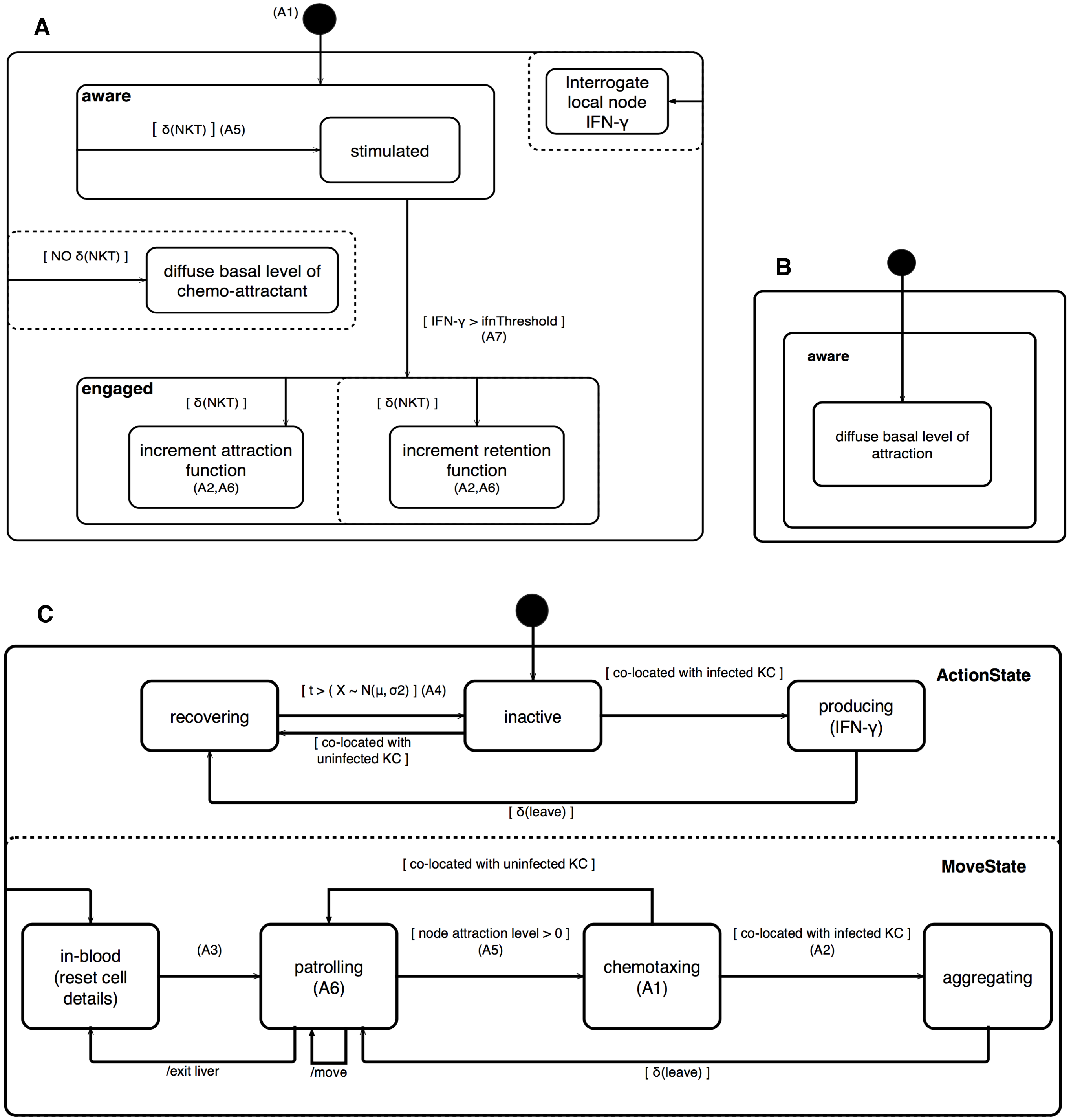

Figure S2. Related to Figure 2. State-diagrams for Kupffer cell and NKT cell behaviors.
(A) Infected Kupffer Cell; (B) uninfected Kupffer Cell; (C) NKT cell. See State Transition Diagrams in Supplemental Experimental Procedures.

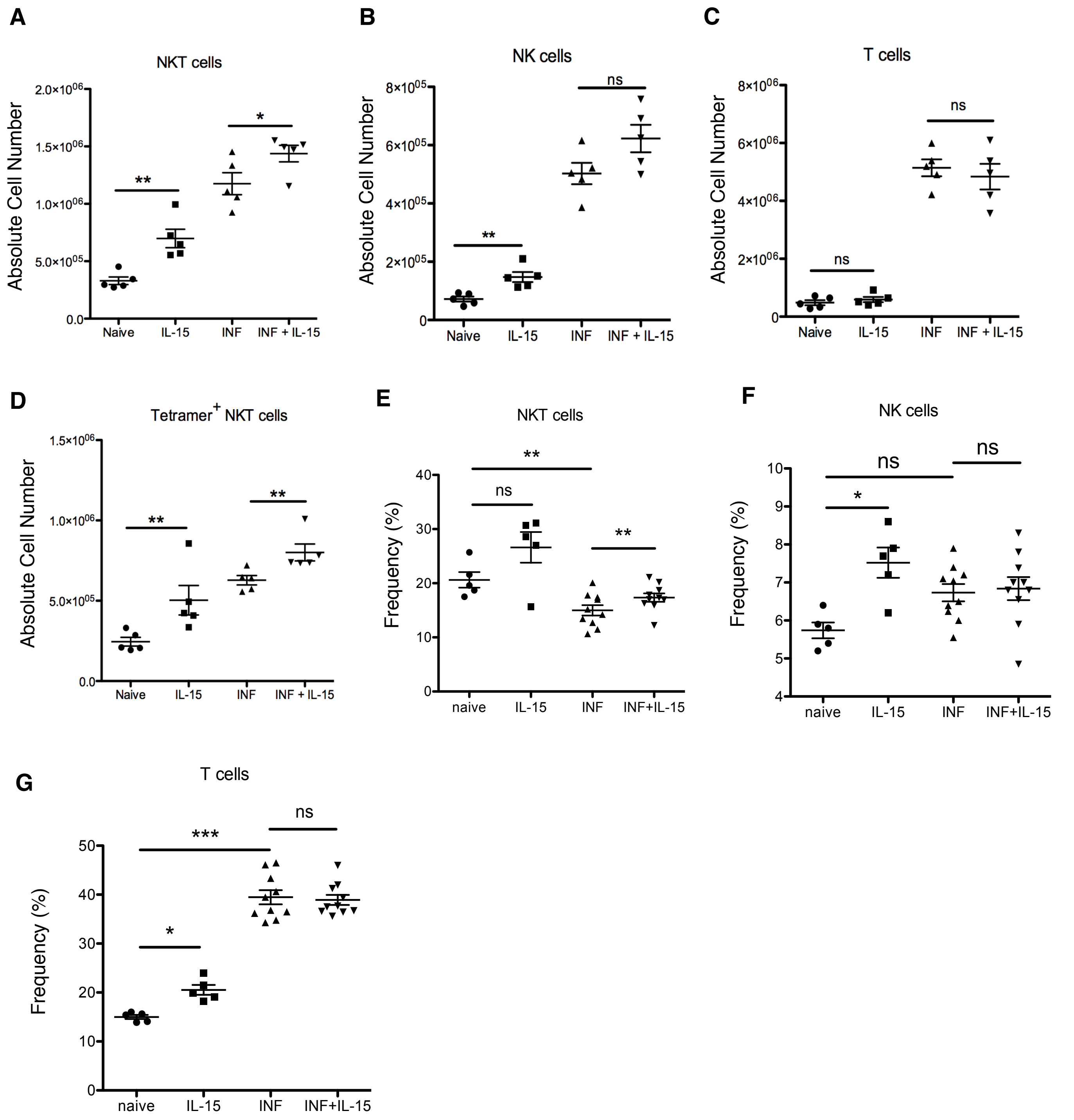

Figure S3. Related to Figure 4. IL-15 Promotes NKT Cell Expansion.
(A) Absolute cell numbers for NKT, (B) NK and (C) T cells, and (D) CD1d tetramer+ NKT cells for naïve, IL-15 treated naïve (IL-15), infected (INF) and IL-15 pre-treated infected (INF+IL-15) mice. (E) Relative frequency of NKT, (F) NK and (G) T cells for naïve, IL-15 treated naïve (IL-15), infected (INF) and IL-15 pre-treated infected (INF+IL-15) mice. Results are depicted as mean ± SEM of 5 mice per group. *P≤0.05, **P≤0.01 paired Students t-test.

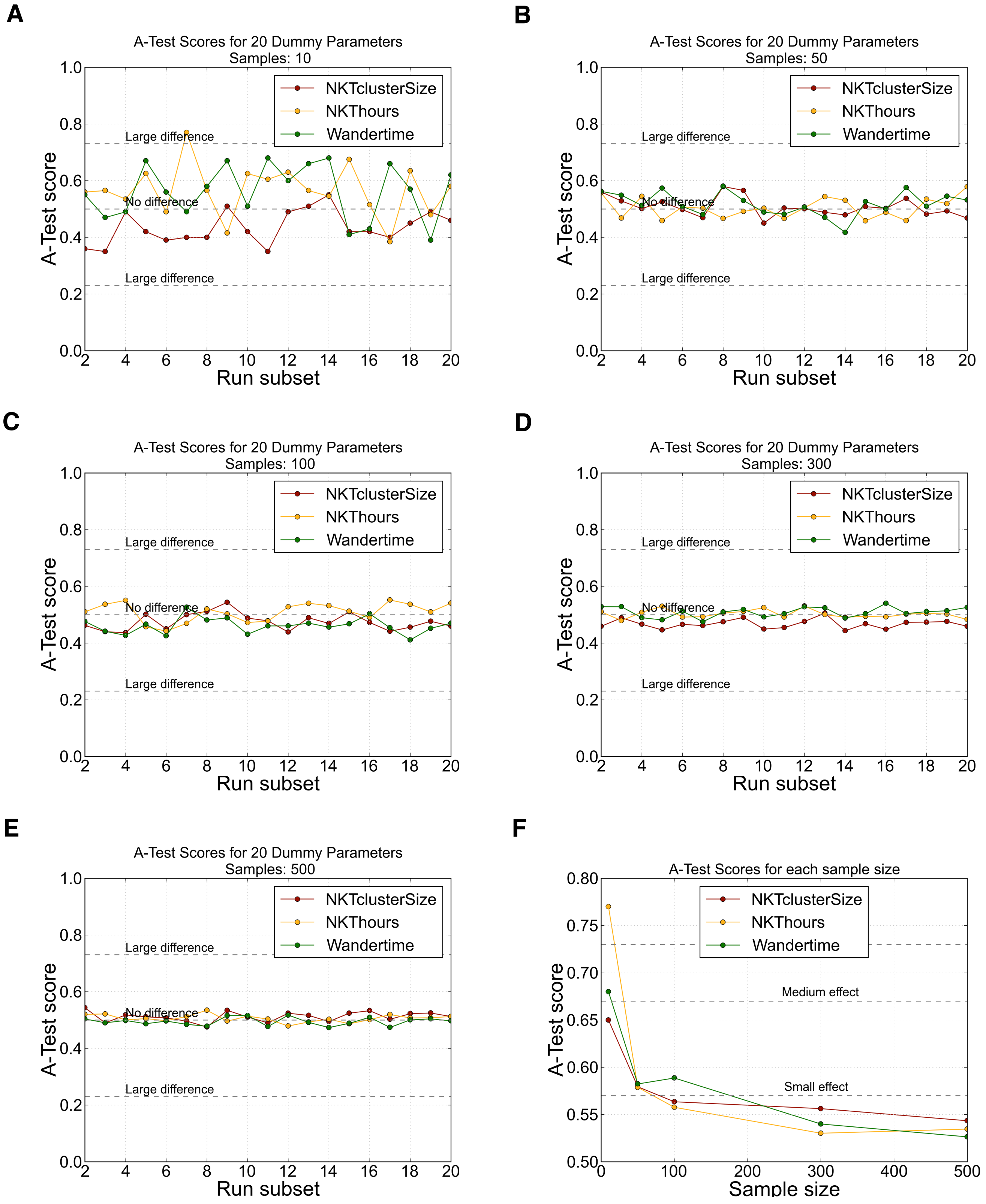

Figure S4. Related to Figure 2. Aleatory Analysis Determines a Minimum of 300 Simulation Runs per Experiment. A-test (Vargha and Delaney, 2000) scores for three simulation output measures across sample sizes of (A) 10, (B) 50, (C) 100, (D) 300 and (E) 500, illustrating that greater than 300 simulation runs are needed to capture the variation in output results and mitigate uncertainty, summarized in (F).

Supplemental Tables

Biological Parameters	Value	Source
NKT cell velocity in the sinusoids	10-20μm/minute	(Geissmann et al., 2005)
NKT cell numbers in a section of mouse liver lobule	$\sim$49($\sim$1:3 ratio with KCs)	Derived from (Lee et al., 2010) and unpublished data
Kupffer cell numbers per gram	14-20 x 10 ^{6}/g	(Bouwens et al., 1986)
Infected KCs at 2 hours	$\sim$23%	Unpublished data
KCs per lobule section	$\sim$146	Unpublished data
Non-branched segment length	43.1 $\pm$ 2.25 μm	(Höhme et al., 2010)
Mean branching angles of sinusoids	32.5° $\pm$ 11.2°	(Höhme et al., 2010)
KC spatial distribution	Percentages:<br>Periportal (PP) - 43<br>Midzonal (MZ) - 28<br>Centrilobular (CV) - 29<br>Ratios: 4(PP), 3(MZ), 2(CV)	(Bouwens et al., 1986; Sleyster and Knook, 1982)

Table S1. Related to Figure 2. Table of biological parameters used for calibration.

	Domain	Platform	Justification
KC_A1		Infected KCs are infected at initiation of the simulation, t(0).	KCs rapidly phagocytose parasites. We are not investigating conditions pre-infection.
KC_A2		Infected KCs can recruit and retain cells.	Unpublished data.
KC_A3		Uninfected KCs can only recruit cells.	Unpublished data.
KC_A4	KCs are immobile	KCs have no movement rules	(Lee et al., 2010)
KC_A5	KC retention of NKT cells is probabilistic, mediated by SIRP-a - CD47 interactions and local IFN-g concentration.	Cognate interactions occur when two cells are co-located on the same node and are not modeled explicitly. Retention is modeled probabilistically using a function modified by local IFN-g level	(Beattie et al., 2010; Svensson et al., 2005)
KC_A6	NKT produced IFN-g affects KC attractive chemokine production.	Retentive chemokine level increases in-line with local IFN-g level and increases the diffusion distance of attractive chemokine linearly. Retentive chemokine diffusion distance can decay.	Simplification, no experimental data available.
KC_A7	There is a threshold level of stimulation required to activate a KC.	KCs will not transition to an engaged state until a threshold level of IFN-g is reached.	To facilitate implementation, no experimental data available.
NKT_A1	NKT cells respond to chemo- attractant	NKT cells under chemotaxis, when presented with two or more attraction gradients, will choose a direction based on attractive chemokine strength.	To facilitate implementation, no experimental data available.
NKT_A2	NKT cells interact with KCs	NKT cells will interact with the first infected KC they encounter whilst in chemotaxis.	To facilitate implementation, no experimental data available.
NKT_A3		The NKT cell population of the lobule section remains constant; cells exiting the environment will enter as new cells via an entry point.	
NKT_A4	NKT cells are capable of becoming anergic	NKT cells are refractive to stimulation and take a period of time to recover after stimulating and leaving a KC.	(Iyoda et al., 2010)
NKT_A5		NKT cells will respond immediately to a chemokine signal.	To facilitate implementation, no experimental data available.
NKT_A6	NKT cells can walk the sinusoids and switch direction probabilistically NKT cells perform a random walk of the tree- node structure, and a probability governs their	NKT cells perform a random walk of the tree-node structure, and a probability governs their ability to turn in the sinusoids at random.	(Geissmann et al., 2005)
C_A1	Attractive chemokines flow in the same direction as blood would.	Attraction diffuses downstream of infected KCs towards the central vein.	Chemotaxis in 3D environments is poorly understood (Haessler et al., 2011)
C_A2		Strength of attractive chemokine is a function of distance from source, calculated using a simplified Ficks Law of diffusion.	To facilitate implementation.
C_A3		Chemokine growth is calculated using a sigmoid function.	To facilitate implementation, no experimental data available.

Table S2. Related to Figure 2. Modelling Assumptions. Statement of assumptions made regarding the underlying biological domain (domain) and how we have abstracted this information in the engineered simulation (platform). Assumptions labeled KC_ relate to Kupffer Cells, NKT_ to NKT Cells, and C_ to chemokines. Assumptions make it possible to model when data is limited or there is a gap in understanding or the literature.

Parameter		Value	Units	Description	Source
Simulation	p_numInfectedKCs	33	cells	Number of infected kupffer cells in a π*(284μm)^2 sectional area of sinusoid.	Calibrated to unpublished data
	p_numUninfected	113	cells	Number of uninfected kupffer cells in a π*(284μm)^2 sectional area of sinusoid.	
	p_numNKTs	49	cells	Number of NKT cells in a π*(284μm)^2 sectional area of sinusoid.	Calibrated to unpublished data and (Lee et al., 2010)·(Geissmann et al., 2005)
Chemokine	p_chemoAttract	43200	iterations	Stimulation time required to reach maximum attractive chemokine concentration.	No biological equivalent; explored and chosen through parameter sensitivity analysis
	p_chemoRetain	172800	iterations	Interaction time required to reach maximum retentive chemokine concentration.	
	p_chemoIFN	172800	iterations	Interaction time required to reach activate infected KCs.	

Parameter		Value	Units	Description	Source
NKT cell	p_turnProb	0.005	probability	Probability that an NKT cell will reverse direction in the sinusoids.	No biological equivalent; explored and chosen through parameter sensitivity analysis.
	p_moveMin	3	iterations	Value given to link simulation iterations to NKT cell velocity.	Calibrated to published NKT cell speeds from (Geissmann et al., 2005)
	p_moveMax	6			
	p_anergicItns	3600	iterations	Time in iterations for an NKT cell to remain unable to stimulate a KC.	No biological equivalent; explored and chosen through parameter sensitivity analysis.
	p_escapeItns	600	iterations	Time in iterations for an NKT cell to escape the influence of KC produced chemo-attractant.	
	p_leaveProb	0.000265306	probability	The probability of an interacting NKT cell leaving the location of an infected KC.	
	p_minLeaveProb	0.00005	probability	To guard against the probabilistic tipping point whereby retention causes cells to never leave.	
	p_chemoIFN	172800	iterations	Interaction time required to reach maximum attractive chemokine concentration.	
Kupffer cell	p_chemoDist	20	distance(nodes)	Starting diffusion distance for attractive chemokine.	No biological equivalent; explored and chosen through parameter sensitivity analysis.
	p_ratioCV	0.1	percentage	Ratio of infected cells in the CV region of the lobule section	Bouwens et al., 1986. Sleyster et al., 1982.
	p_ratioMZ	0.25	percentage	Ratio of infected cells in the MZ region of the lobule section	
	p_ratioPP	0.65	percentage	Ratio of infected cells in the PP region of the lobule section	
	p_maxDist	200	distance(nodes)	Maximum diffusion distance for attractive chemokine.	Calibrated to twice reported max(Weber et al., 2013)
	p_ifnThreshold	0.999	threshold	Threshold value of IFN-γ required to activate a KC. Chemokine function f(x)→1, therefore a threshold is required.	No biological equivalent

Table S3. Related to Figure 2. Simulation Parameters. Summary of the simulation parameters, descriptions of their purpose, values and any data sources for parameters relating to cell numbers and chemokine functions. All estimated values are based on a comprehensive sensitivity analysis for parameters that have unknown or no clear biological value.

Supplemental Movies

Movie S1. Related to Figure2. Sinusoid structure generation algorithm. Execution of the sinusoid structure generation algorithm at 8x speed. Drain node (cyan), entry nodes (green) and sinusoids (red).

Supplemental Experimental Procedures

We assume that our sinusoid network exists in a quasi-2D space (we can consider this as a slice through a 3D lobule). We also assume that the lobule structure is roughly hexagonal with a single central vein in the centre and six portal triad areas placed at roughly regular intervals around the central vein. The flow of blood borne cells is assumed to be from portal triads to the central vein, so in the algorithmic description below the central vein is termed a drain node, and the portal triad regions deemed entry nodes.

Algorithm 1 describes how the nodes and edges of the sinusoid network are generated, whilst Algorithm 2 describes how the overall sinusoid network (the lobule) is generated using Algorithm 1. Höhme et al. (2010) provide us with the following statistics that guide Algorithm 2:

- Average length between central vein and portal triad = 284μm;
- Average length of a non-branched sinusoid = 43.1μm;
- Average angle between branching sinusoids = 32.5°.

Algorithm 1: Sinusoid branch generation

1. A potential new node (μ) is generated 1μm from the current node (δ)
   (a) If we are within range of an attracting node (α) then μ is generated in the direction of α (see Figure S4(B)).
   (b) Otherwise μ is generated based on our current direction with a small random adjustment (see Figure S4(A)).
2. Create a new edge between δ and another node:
   (a) If the line between δ and μ intersects another edge in the sinusoid network, then connect δ to the closest existing node (see Figure S4(D)).
   (b) Otherwise connect δ and μ (see Figure S4(C)).
3. Repeat Steps 1 and 2 until an intersection is detected.

Algorithm 2: Sinusoid network (lobule) generation

1. A drain node (representing the central vein) is placed in the centre of the 2D space, surrounded by six entry nodes (representing the locations of portal triads) in an irregular hexagon formation (see Figure 2(Ai)). The exact location of the entry nodes is determined stochastically.
2. For each of the six entry nodes a sinusoid branch is grown (see algorithm 1) from the entry node towards the attracting drain node (see Figure 2(Aii)).
3. An additional set of entry nodes is created for each original entry node and aligned with the original node. These additional nodes represent additional sources of blood supply coming out of the portal triad (see Figure 2(Aiii)).
4. For each of the new entry nodes a sinusoid branch is grown (see algorithm 1) from the entry node towards the existing sinusoid structure (see Figure 2(Aiv)).
5. Additional sinusoids are created to connect existing sinusoids (see Figure 2(Av)).
   - Select the longest sinusoid in the structure
   - Select a node in the longest sinusoid and grow a sinusoid (see algorithm 1) to either the left or right at an angle drawn from a normal distribution with a mean of 32.5°.
   - Repeat until the mean sinusoid length of the entire structure reaches 43.1μm.

Cell Attraction Dynamics

If we assume an infected KC diffuses chemokine to a downstream node α, chemokine strength at α is a function of distance:

$$\lambda / \delta$$

Where:

λ is the chemokine strength at the infected node.
δ is the distance in nodes between the KC and α.

Uninfected KCs are unable to modify their attraction diffusion distance from the parameterized minimum (Scenario A only), whereas infected-KC attractive chemokine diffusion distance is variable between a minimum and maximum distance. That distance is calculated as a function of the current level of attractive chemokine at the source KC location:

$$\delta = | \lambda \cdot (\delta\text{max} - \delta\text{min}) |$$

Where:

λ is the chemokine strength at the infected node.
δmax is the maximum parameterized diffusion distance.
δmin is the minimum parameterized diffusion distance

The function is floored to the nearest integer and that is used as the updated diffusion distance.

Cell Retention Dynamics

The equation governing NKT cell retention is:

$$\delta\text{nktleave} = \delta\text{leave} \ \ (\phi \cdot \delta\text{leave})$$

Where:

δnktleave is the probability of an NKT cell leaving an infection site.
δleave is the maximum parameterized retention probability.
ϕ is the level of retentive chemokine at the infected node.

As the calculated probability will approach zero given suitable conditions, a minimum retention probability is parameterized to ensure that KCs do not become so retentive that NKT cells are then incapable of leaving.

State-Transition Diagrams

Figure S2 depicts state-transition diagrams using the Unified Modelling Language (UML). These diagrams are the engineering specific (platform) ones used to create the simulator. To improve clarity, various annotations are added to convey information relevant to our modelling context. Arrows denote transitions between states. Square brackets ([]) denote guards for a transition, conditions that must be met before a transition can occur. Dashed lines denote states or behaviours that occur concurrently. Where an assumption number is stated on a diagram, denoted by (An), refer to the relevant cell assumption table. The $\delta()$ notation denotes an interaction that might occur probabilistically, for example a cell-cell interaction.

- **Infected Kupffer Cells** (Figure S2(A)): begin in an aware state and have a minimum level of attractive chemokine and minimum diffusion distance of attraction. If the level of cell-local interferon-gamma produced by NKT cells reaches a threshold (ifnThreshold), infected KCs become engaged. When infected KCs are engaged, if there is sustained interaction with NKTs, indicated by δ(NKT), they will increase their level of attraction and retention.

- **Uninfected Kupffer Cells** (Figure S2(B)): always in an aware state and only diffuse the minimum level of attractive chemokine. They do not interact with NKTs by any other means.

- **NKT cells** (Figure S2(C)): have two state types, ActionStates and MoveStates. The initial ActionState is inactive. If an infected KC is encountered the NKT will begin producing interferon- until probabilistically leaving mediated by the retention level of the KC. If NKT cells encounter uninfected KCs, they will transition to the recovering state. NKTs leave the recovering state after a time sampled from a normal distribution, this time is significantly shorter for NKTs that previously left uninfected KCs, and hence havent been in the producing state. The default NKT cell MoveState is patrolling. Upon sensing a level of attraction, NKT cells will transition to a chemotaxing state. Interaction with an infected KC will cause the NKT to switch to an aggregating state; alternatively an uninfected KC encounter will lead back to a patrolling state. Aggregating NKT cells can transition to patrolling behavior probabilistically. Should NKT cells exit the liver environment, if the entry condition is satisfied, they will emerge from an environment entry point, effectively as another cell.

Parameterizing and Calibrating the Simulation

A full summary of the biological data available that was used to calibrate the simulation is listed in Table S1, though these are merely the domain specific parameters, and a number of implementation specific parameters are required in order to abstract domain behaviors into executable computer code. A good example of an implementation specific parameter relates to NKT cell speed. NKT cells traverse the sinusoids at 10-20m/minute. In the simulation, this corresponds to 10-20 nodes/minute. Our simulation iterations are in seconds, so for a cell to travel at a maximum speed of 20 nodes/minute it would have to move every 3 simulation iterations (p_moveMin), and a minimum speed of 10 nodes/minute every 6 simulation iterations (p_moveMax). Rather than have individual cell speed remain constant, we allow it to be dynamic within the published biological range. We calculate, probabilistically between p_moveMin and p_moveMax, the number of iterations a cell will remain stationary before its next move. This allows individual cells to speed up and slow down dynamically, yet maintains a normal distribution of cell speeds across the population and within the biologically specified range. The entire list of baseline simulation parameters can be viewed in Table S3. Several parameters have no biological equivalent though are fundamental for the implementation of many behaviors. We performed parameter sensitivity analysis (SA) in order to determine which parameters the simulation is extremely sensitive to, and to establish baseline parameter values. Finally SA allows us to ensure we are always interpreting our results with the knowledge that particular extreme parameter combinations might influence those results.

Each simulated experiment is run across 10 separate structures in order to approximate variance across the set. For each parameter value investigated (or combination of parameter values) per experiment, 500 simulation runs are performed, this number chosen after performing aleatory uncertainty analysis on the simulator. Aleatory analysis can be used to determine the minimum number of replicates runs required to both mitigate the effects of stochasticity on simulation output, and to generate results that cover a representative spectrum of possible system behaviours (Alden et al., 2013). Fig. S3f shows that 300 simulation runs per parameter combination are sufficient to have acceptable uncertainty (small variance between identical sample sizes), though we perform 500 in order to strike a balance between further reducing A-test effect size (Vargha and Delaney, 2000) and maintaining tractable simulated-experiment execution times.

Supplemental References

Haessler, U., Pisano, M., Wu, M., and Swartz, M.A. (2011). Dendritic cell chemotaxis in 3D under defined chemokine gradients reveals differential response to ligands CCL21 and CCL19. Proceedings of the National Academy of Sciences of the United States of America 108(14), 5614-5619.

Iyoda, T., Ushida, M., Kimura, Y., Minamino, K., Hayuka, A., Yokohata, S., Ehara, H., and Inaba, K. (2010). Invariant NKT cell Anergy is Induced by a Strong TCR-Mediated Signal Plus Co-stimulation. International Immunology 22(11), 905913.

Vargha, A., and Delaney, H. D. (2000). A Critique and Improvement of the "CL" Common Language Effect Size Statistics of McGraw and Wong. Journal of Educational and Behavioral Statistics 25(2), 101132.
